# The Effect of Chinese Herbal Medicine Combined With Western Medicine on Vascular Endothelial Function in Patients With Hypertension: A Systematic Review and Meta-Analysis of Randomized Controlled Trials

**DOI:** 10.3389/fphar.2020.00823

**Published:** 2020-06-16

**Authors:** Weiquan Ren, Miyuan Wang, Jiangquan Liao, Lingling Li, Deshuang Yang, Ruiqi Yao, Li Huang

**Affiliations:** ^1^Beijing University of Chinese Medicine, Beijing, China; ^2^China–Japan Friendship Hospital, Beijing, China

**Keywords:** blood pressure, hypertension, Traditional Chinese Medicine, systematic review, meta-analysis

## Abstract

**Objective:**

Vascular endothelium plays a fundamental role in regulating endothelial dysfunction, resulting in structural changes that may lead to adverse outcomes of hypertension. The aim of this study was to systematically evaluate the effect of a combination of Chinese herbal medicine (CHM) and Western medicine on vascular endothelial function in patients with hypertension.

**Methods:**

We systematically searched the literature for studies published in Chinese and English in PubMed, Embase, Cochrane Central Register of Controlled Trials, Chinese Biomedical Literature Database, China Knowledge Resource Integrated Database, Wanfang Data, and China Science and Technology Journal Database. Databases were searched using terms concerning or describing CHM, hypertension, vascular endothelium, and randomized controlled trials. RevMan 5.3.0 was used for data analysis. If the included studies were sufficiently homogeneous, quantitative synthesis was performed; if studies with different sample sizes and blind methods were used, subgroup analyses were performed. GRADEpro was selected to grade the current evidence to reduce bias in our findings.

**Results:**

In this review, 30 studies with 3,235 patients were enrolled. A relatively high selection and a performance bias were noted by risk of bias assessments. Meta-analysis showed that the combination of CHM and conventional Western medicine was more efficient than conventional Western medicine alone in lowering blood pressure (risk ratio, 1.21; 95% CI, 1.16 to 1.26) and increasing nitric oxide (95% CI, 1.24 to 2.08; *P <* 0.00001), endothelin-1 (95% CI, −1.71 to −1.14; *P <* 0.00001), and flow-mediated dilation (95% CI, 0.98 to 1.31; *P <*0.00001). No significant difference was observed between the combination of CHM and conventional Western medicine and conventional Western medicine alone for major cardiovascular and cerebrovascular events. CHM qualified for the treatment of hypertension. The GRADEpro presented with low quality of evidence for the available data.

**Conclusion:**

CHM combined with conventional Western medicine may be effective in lowering blood pressure and improving vascular endothelial function in patients with hypertension. To further confirm this, more well-designed studies with large sample sizes, strict randomization, and clear descriptions about detection and reporting processes are warranted.

## Introduction

Cardiovascular disease (CVD) is the primary cause of death- and disability-adjusted life-years (DALYs) worldwide and is responsible for 12.9 million deaths and 0.3 billion DALYs each year (Lozano et al., 2012; Murray et al., 2012), for which high blood pressure (BP) is the strongest risk factor (Writing Group et al., 2016). In 2015, 1.13 billion individuals were reported to have CVDs worldwide (Collaboration, 2017). The vascular endothelium plays a fundamental role in regulating the vascular tone and structure as well as endothelial dysfunction, resulting in structural changes that may lead to adverse outcomes of hypertension (Juonala et al., 2006). Well-maintained endothelial function and integrity are of great significance in numerous conditions, including hypertension, inflammatory and cardiovascular diseases, and their risk factors (Sattar et al., 2003; Touyz and Briones, 2011).

Antihypertensive agents, including diuretics, beta-adrenergic blocking agents (β-blockers), angiotensin-converting enzyme inhibitors (ACEIs), angiotensin II receptor blockers (ARBs), and calcium channel blockers (CCBs) are mostly used in the treatment of CVDs. However, around half of the patients are incapable of effectively controlling their BP by drug therapy owing to the associated cost, adverse reactions, and complications (Shaw et al., 1995). Behavioral interventions, such as exercise, weight loss, and salt intake casn control and help lower the BP, but these are hard to comply with.

Traditional Chinese Medicine (TCM) has proven to be an important part of complementary and alternative medicine (CAM) because of efficacious clinical practice in China, although its mechanism remains unclear (Harris et al., 2012). Many studies have shown that hypertension can be effectively managed by Chinese herbal medicine (CHM), acupuncture, and tai chi (Shih et al., 2005; Wang et al., 2007; Yin et al., 2007; Kim and Zhu, 2010). In clinics, patients with essential hypertension are commonly treated with CHM combined with antihypertensive agents. However, there is no systematic summary available of the RCTs examining the efficacy of CHM plus antihypertensive drugs (CPAD) for vascular endothelial function in patients with essential hypertension. Thus, to critically assess the efficacy of CPAD for essential hypertension, we performed this systematic review.

## Methods

This meta-analysis was carried out and reported following the Preferred Reporting Items for Systematic Reviews and Meta-Analyses (PRISMA) (Moher et al., 2010). A protocol has been registered in PROSPERO for this review (registration number: CRD42019140743).

### Inclusion Criteria

#### Participants

In this meta-analysis, we did not restrict based on patient's age, gender, course of disease, case source, nationality, or race. In the original literature, the definition of hypertension is consistent with past guidelines (systolic BP [SBP] ≥140 mmHg or diastolic BP [DBP] ≥90 mmHg) (Hypertension Alliance (China) et al., 2019). The exclusion criteria included (a) subjects with hypertension complicated by other serious CVDs, hepatic failure, or renal failure, (b) secondary hypertension, (c) gestational hypertension, or (d) isolated systolic hypertension.

#### Intervention

According to the 2015-edition of the Pharmacopoeia of the People's Republic of China, compiled by the China Food and Drug Administration, CHM was defined as herbal agents and materials derived from the botanical herbal products, minerals, and animal sources. CHMs were prepared into various forms such as decoctions, tablets, pills, powders, granules, capsules, oral liquids, and injections. Based on the TCM pattern identification and treatment by experienced doctors, usually a compound formula consists of two or more herbs to obtain a synergistic effect under certain circumstances. All types of CPADs without considering the dose, method of dosing, composition of the formula, or time of drug administration were compared with the Western medicine. The comparisons included in this study were as follows: (a) CHM combined with CCB vs CCB; (b) CHM combined with ACEI vs ACEI; (c) CHM combined with ARB vs ARB; (d) CHM combined with diuretic vs diuretic; (e) CHM combined with multiple antihypertensive drugs (CCB/ACEI/ARB/diuretic/β-blocker) vs multiple antihypertensive drugs.

#### Control

As mentioned above, the control could be CCB, ACEI, ARB, diuretic, β-blocker alone, or multiple antihypertensive drugs.

#### Outcomes

Primary outcomes were 24 h ambulatory BP monitoring (24 h-SBP and 24 h-DBP), SBP, DBP, and therapeutic effectiveness with reference to the standards of Chinese Medicine Clinical Research of New Drugs Guiding Principles. Marked effectiveness was considered as: DBP decreased ≥20 mmHg but did not reach normal level; or 10 mmHg ≤ DBP decrease < 20 mmHg and reached normal level; BP decrease <10 mmHg with normal level; or 10 mmHg ≤ DBP decrease < 20 mmHg but did not reach normal level; or SBP decrease ≥ 30 mmHg but did not reach normal level; ineffectiveness: DBP decreased < 20 mmHg and did not reach normal levels. Secondary outcomes were nitric oxide (NO), endothelin-1 (ET-1), flow-mediated dilation (FMD), vascular endothelial growth factor (VEGF), high-sensitivity C-reactive protein (hs-CRP), angiotensin II (Ang II), von Willebrand factor (vWF), and transforming growth factor β-1 (TGFβ-1). Among them, NO protects the vascular endothelial function, whereas increase in ET-1, VEGF, hs-CRP, Ang II, vWF, and TGFβ-1 adversely affects vascular endothelial function. The decrease in FMD indicates an impairment of the vascular endothelial function.

#### Study Type

Randomized controlled trials (RCTs) that combined CHM with antihypertensive drugs to treat hypertensive patients regardless of blinding, were included. To minimize publication bias, there was no restriction on language and time.

### Literature Searches

We conducted a systematic search of studies published in Chinese and English using the following databases: PubMed, Embase, Cochrane Central Register of Controlled Trials(CENTRAL), Chinese Biomedical Literature Database (CBM), China Knowledge Resource Integrated Database (CNKI), Wanfang Data, and China Science and Technology Journal Database (VIP). These databases were searched from inception to April 2019. Terms related to CHM, hypertension, and RCTs were searched in these databases. The search was not restricted by language or publication dates. The search strategy that suited each database was as follows: (“Hypertension” OR “High Blood Pressure”) AND (“Endothelium, Vascular” OR “Endothelium”) AND (“Medicine, Chinese Traditional” OR “Drugs, Chinese Herbal” OR “Traditional Chinese Medicine” OR “Chinese medicine” OR “Chinese medica” OR “Chinese drug” OR “Chinese patent medicine” OR “CHM” OR “Herbal medicine” OR “Chinese Herbal Drug” OR “Chinese Plant Extract”).

**Figure 1:**
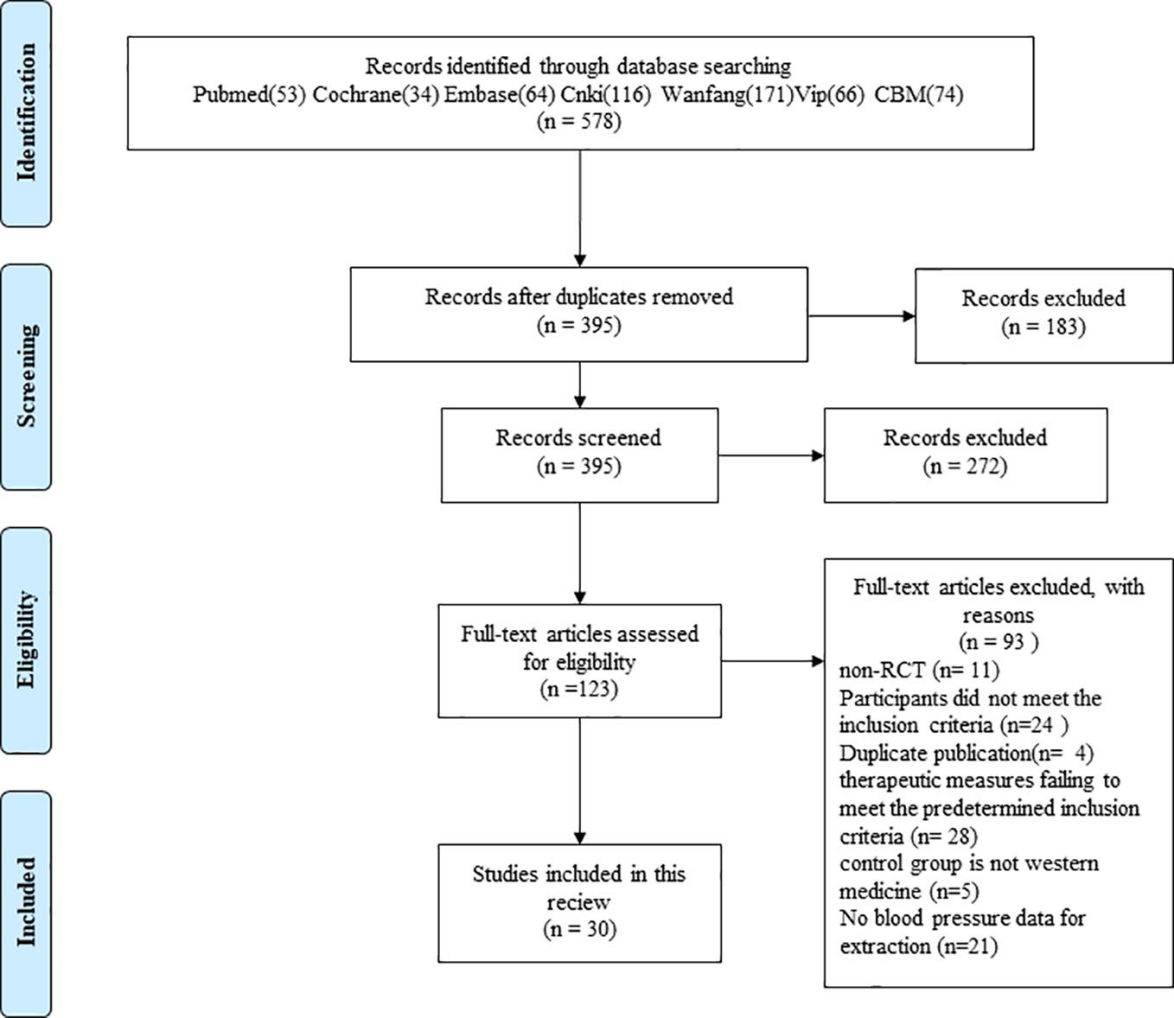
Flow diagram of literature search and study selection.

After removing duplicates, two investigators independently screened the titles and abstracts of the articles obtained *via* initial search for relevance. Abstracts that did not meet our criteria were excluded, and few abstracts with insufficient information regarding the inclusion criteria were further reviewed. The full texts of the remaining results were further determined by the same investigators but by blinding to each other's review. All disagreements were resolved by consensus. The flow chart of the study selection is shown in **Figure 1**.

### Data Extraction

Data were extracted in duplicate by two investigators independently and were inputted to a dedicated database. The data extracted from each article included basic information (study ID, document type, author, and publication year), participant's demographic details (sample size, age, and sex), diagnostic criteria, inclusion and exclusion criteria, study drug and control treatment, outcomes, fall outs, follow-up duration, and outcomes. Disagreements regarding the extracted data were settled by a third reviewer (AAN).

### Risk of Bias Assessment

We used the Cochrane Collaboration “risk of bias” (ROB) tool for ROB assessment. Referring to the Cochrane Handbook criteria for assessing ROB in the ROB assessment tool, two investigators independently assessed the methodological quality of the included studies using RevMan 5.3.0. Following the handbook, the assessment of ROB was graded as low, unclear, or high based on the following seven aspects: random sequence generation (selection bias), allocation concealment (selection bias), blinding of participants and personnel (performance bias), blinding of outcomes assessment (detection bias), incomplete outcomes data (attrition bias), selective reporting (reporting bias), and other biases. All differences during ROB assessment were resolved by consensus.

### Statistical Analyses

RevMan 5.3.0 was used to analyze the results of this study. For binary data, estimates were described as relative risk (RR) and 95% confidence interval (CI). For continuous data, the weighted mean difference (MD) or standard mean difference (SMD) and 95% CIs were calculated. Only complete case data were selected for further analysis. Heterogeneity between the studies in effect measures was assessed using both the chi-squared test and the *I^2^* statistic (Higgins et al., 2003) with an I*^2^* value >50%, indicative of substantial heterogeneity. Sufficiently homogeneous distribution allowed the use of quantitative synthesis in both statistics and clinic. When the *I^2^* value was lower than 50% and *P >*0.10, a fixed-effect model was adopted; otherwise, a random-effect model was suitable.

Because of significant heterogeneity in primary outcomes, studies with different sample sizes and bindings were subjected to separate subgroup analyses. As few results of the subgroup analysis revealed low methodological quality of the included studies and significant positive results, no further sensitivity analyses were performed.

As the least number of studies in each project was 10, a funnel plot was drawn to detect publication bias. To minimize bias in our findings, we selected the online software GRADEpro to summarize findings for outcomes to evaluate the available evidence. The assessment included biases including risk, inconsistency (heterogeneity), indirect, imprecision, and publication biases; each evidence was graded as very low, low, moderate, or high.

## Results

### Literature Screening

Herein, we have described the literature retrieval process. A total of 578 potentially relevant articles from seven electronic databases were retrieved after the literature search. After removal of duplicates, 395 articles were identified. After going through the titles and abstracts, 272 articles that were case reports, case series, reviews, and animal studies irrelevant to hypertension were excluded. After reading the full text of the remaining 123 articles, 93 studies were further removed for at least one of the following reasons: no RCT (n = 11); participants failing to meet the inclusion criteria (n = 24); duplicates (n = 4); therapeutic measures failing to meet the predetermined inclusion criteria (n = 28), no Western medicine used in the control group (n = 5), and no BP data for extraction (n = 21). Thirty articles in accordance with the inclusion criteria were identified.

### Characteristics of Included Studies

The enrolled 30 articles were published between 2010 and 2019, in which all studies related to comparison of CPAD group vs antihypertensive drug group (Control group). In the CPAD group, the standard, type, and dosage of antihypertensive drugs used were identical to those in the control group.

In total, 3,235 patients were randomly divided into a CPAD group and a control group, all of whom were from China, including 1,388 women. The average age of the participants ranged from 36.0 to 77.3 years. All trials could be accessed *via* full texts. Treatment duration lasted 4–24 weeks, and most of them lasted 8 weeks (11/30, 37%). The primary outcomes measure was reported. Among them, a 24 h ambulatory blood pressure monitoring was reported in seven studies (Xu et al., 2010; Zhu et al., 2010; Wu et al., 2014; Weng and Lin, 2015; Teng et al., 2016; Cao et al., 2019; Sun, 2019), SBP and DBP in 24 (Xu et al., 2010; Gao et al., 2012; Qian et al., 2013; Shen et al., 2013; Zhu et al., 2013; Ou and Li, 2014; Lu et al., 2015; Zhang, 2015; Bian, 2016; Feng et al., 2016; Sheng and Wang, 2016; Wang et al., 2016; Wu et al., 2016; Zheng et al., 2016; Zeng W. Y. et al., 2017; Zeng Z. C. et al., 2017; Gao and Li, 2017; Li, 2017; Li et al., 2017; Ruan et al., 2017; Han et al., 2018; Liu et al., 2018; Luo, 2018; Shi et al., 2018), treatment efficiency in 16 (Xu et al., 2010; Shen et al., 2013; Zhu et al., 2013; Ou and Li, 2014; Lu et al., 2015; Bian, 2016; Sheng and Wang, 2016; Teng et al., 2016; Wang et al., 2016; Zheng et al., 2016; Gao and Li, 2017; Ruan et al., 2017; Zeng W. Y. et al., 2017; Liu et al., 2018; Luo, 2018; Sun, 2019), NO in 18 (Xu et al., 2010; Zhu et al., 2010; Qian et al., 2013; Wu et al., 2014; Zhang, 2015; Bian, 2016; Feng et al., 2016; Sheng and Wang, 2016; Teng et al., 2016; Wang et al., 2016; Zheng et al., 2016; Gao and Li, 2017; Li et al., 2017; Zeng Z. C. et al., 2017; Han et al., 2018; Liu et al., 2018; Shi et al., 2018; Cao et al., 2019), ET-1 in 19 (Xu et al., 2010; Zhu et al., 2010; Qian et al., 2013; Wu et al., 2014; Zhang, 2015; Bian, 2016; Feng et al., 2016; Sheng and Wang, 2016; Teng et al., 2016; Wang et al., 2016; Zheng et al., 2016; Gao and Li, 2017; Li et al., 2017; Zeng W. Y. et al., 2017; Zeng Z. C. et al., 2017; Han et al., 2018; Liu et al., 2018; Shi et al., 2018; Cao et al., 2019), FMD in 8 (Gao et al., 2012; Shen et al., 2013; Weng and Lin, 2015; Zhang, 2015; Wu et al., 2016; Zheng et al., 2016; Zeng W. Y. et al., 2017; Sun, 2019), VEGF in 5 (Ou and Li, 2014; Lu et al., 2015; Li, 2017; Ruan et al., 2017; Luo, 2018). hsCRP), hs-CRP in 5 (Gao et al., 2012; Qian et al., 2013; Zhu et al., 2013; Bian, 2016; Wang et al., 2016), Ang II in 3 (Sheng and Wang, 2016; Zeng Z. C. et al., 2017; Sun, 2019), vWF in 3 (Wu et al., 2016; Zheng et al., 2016; Zeng W. Y. et al., 2017), and TGFβ-1 in 2 studies (Ruan et al., 2017; Luo, 2018) (**Table 1**).

**Table 1:** Characteristics of included studies.

Certainty							NO of patients		Effect		Certainty	Importance
No. of studies	Study design	Risk of bias	Inconsistency	Indirectness	Imprecision	Other considerations	CHM combined with west medicine	West Medicine	Relative (95% CI)	Absolute (95% CI)		
24 h-SBP												
7	randomized trials	serious^a^	serious^b^	not serious	not serious	none	408	403	–	SMD 0.85 lower (1.43 lower to 0.26 lower)	⨁⨁◯◯ LOW	CRITICAL
24 h-DBP												
7	randomized trials	serious^a^	serious^b^	not serious	not serious	none	408	403	–	SMD 0.76 lower (1.29 lower to 0.24 lower)	⨁⨁◯◯ LOW	CRITICAL
SBP												
24	randomized trials	serious^a^	serious^b^	not serious	not serious	none	1,259	1,264	–	MD 8.3 lower (10.4 lower to 6.19 lower)	⨁⨁◯◯ LOW	CRITICAL
DBP												
24	randomized trials	serious^a^	serious^b^	not serious	not serious	none	1,259	1,264	–	SMD 0.93 lower (1.13 lower to 0.74 lower)	⨁⨁◯◯ LOW	CRITICAL
NO												
18	randomized trials	serious^a^	serious^b^	not serious	not serious	publication bias strongly suspected^c^	973	969	–	SMD 1.66 higher (1.24 higher to 2.08 higher)	⨁⨁◯◯ LOW	CRITICAL
ET-1												
19	randomized trials	serious^a^	serious^b^	not serious	not serious	none	1,056	1,053	–	SMD 1.42 lower (1.71 lower to 1.14 lower)	⨁◯◯ VERY LOW	CRITICAL
FMD												
8	randomized trials	serious^a^	serious^b^	not serious	not serious	none	418	424		MD 1.14 higher (0.98 higher to 1.31 higher)	⨁⨁◯◯ LOW	CRITICAL

CI, confidence interval; MD, mean difference; SMD, standardized mean difference.

Explanations:

a ano-blinded.

b I2 >50%.

c The funnel plot is asymmetrical.

### ROB Assessment

**Figure 2:**
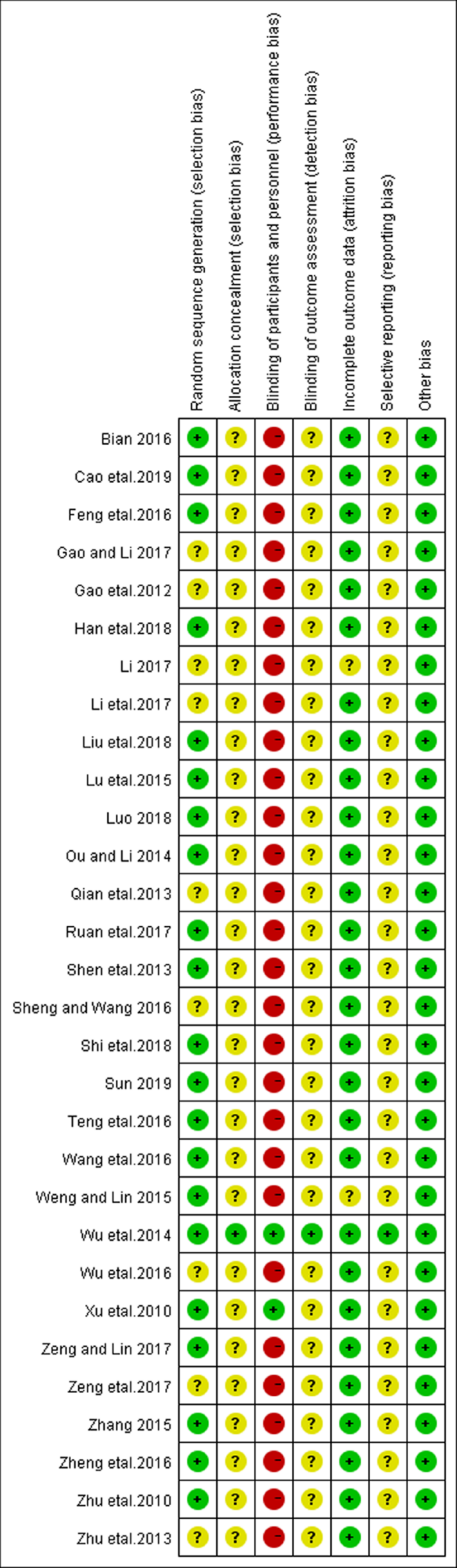
Risk of bias graph.

**Figure 3:**
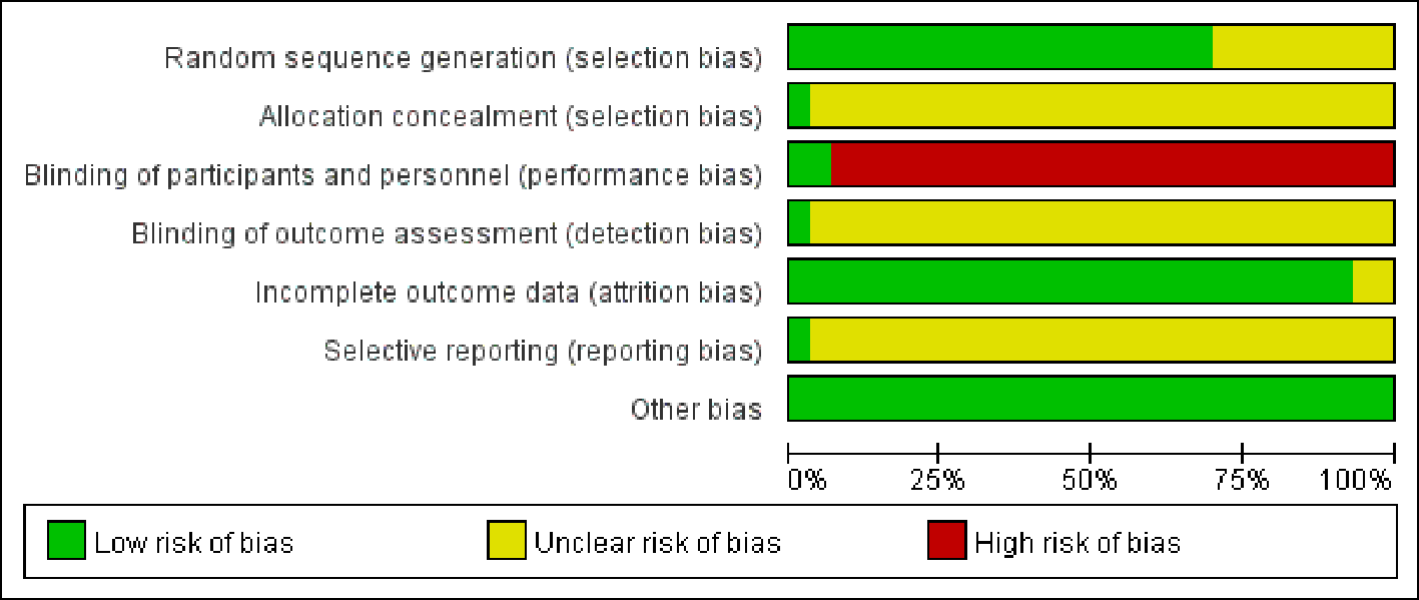
Risk of bias summary: reviewing authors' judgments regarding each risk of bias item for each included study.

Two reviewers independently extracted the data from these included studies and conducted an ROB assessment using the Cochrane Collaboration's tool for ROB assessment. In this systematic review, all 30 trials were reported as RCTs. Of 30, 18 reported the generation of the allocation sequence. Among them, 15 used a random number table (Zhu et al., 2010; Shen et al., 2013; Ou and Li, 2014; Lu et al., 2015; Bian, 2016; Feng et al., 2016; Teng et al., 2016; Wang et al., 2016; Zheng et al., 2016; Ruan et al., 2017; Zeng Z. C. et al., 2017; Han et al., 2018; Liu et al., 2018; Luo, 2018; Shi et al., 2018), two used a randomization list generated with an SAS software package (Wu et al., 2014; Cao et al., 2019) and one used the stratified block randomization (Sun, 2019). Only one trial (Wu et al., 2014) reported allocation concealment. Two trials reported the use of double blinding (Xu et al., 2010; Wu et al., 2014). Three trials reported withdrawals (Gao et al., 2012; Teng et al., 2016; Zheng et al., 2016). No protocols for the included studies were available for us to investigate the selective reporting. We found only one study (Wu et al., 2014) that is included in this review to be at low risk for selective reporting, with the remaining 29 studies being assessed as high risk. No other potential sources of bias were detected. The ROB in the included studies is shown in **Figures 2** and **3**.

### Efficacy Analyses

#### BP

**Figure 4:**
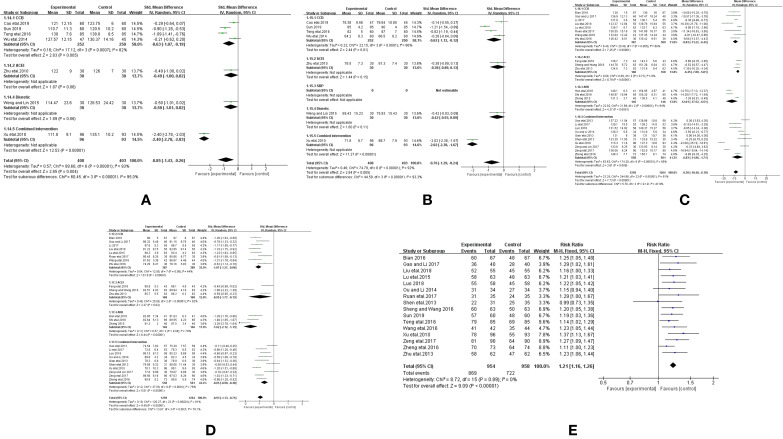
Forest plot of the comparison between CHM combined with conventional Western medicine and conventional Western medicine alone for **(A)** 24 h-SBP, **(B)** 24 h-DBP, **(C)** SBP, **(D)** DBP, and **(E)** therapeutic efficacy.

Seven trials (Xu et al., 2010; Zhu et al., 2010; Wu et al., 2014; Weng and Lin, 2015; Teng et al., 2016; Cao et al., 2019; Sun, 2019) reported the treatment effects on BP, as measured by the 24 h ambulatory BP monitor (24h-SBP and 24h-DBP), including a total of 1,211 patients. We found that 24 h-SBP (*I^2^* = 93%, *P <* 0.00001) and 24h-DBP (*I^2^* = 92%, *P <* 0.00001) were highly heterogeneous; thus, we further conducted a subgroup analysis and selected a random-effects model to classify Western medicines for improving 24 h-SBP into CCB (MD = −0.63, 95% CI [−1.07, −0.19], *P* = 0.005), ACEI (MD = −0.49, 95% CI [−1.00, 0.02], *P* = 0.06), diuretic (MD = −0.5, 95% CI [−1.01, −0.02], *P* = 0.06), and combined intervention (MD = −2.4, 95% CI [−2.78, −2.03], *P* = 0.00001). Meanwhile, Western medicines used to improve 24 h-DBP were also classified into CCB (MD = −0.62, 95% CI [−1.12, −0.12], *P* = 0.01), ACEI (MD = −0.38, 95% CI [−0.89, 0.13], *P* = 0.15), diuretic (MD = −0.42, 95% CI [−0.93, 0.09], *P* = 0.11), and combined intervention (MD = −2.02, 95% CI [−2.38, −1.67], *P* = 0.00001). Compared with Western medicine, combined with CHM based on CCB and combined intervention could remarkably reduce 24 h-SBPand 24 h-DBP, but CHM combined with ACEI and diuretic showed no obvious improvement (**Figures 4A, B**).

Twenty-four trials (Xu et al., 2010; Gao et al., 2012; Qian et al., 2013; Shen et al., 2013; Zhu et al., 2013; Ou and Li, 2014; Lu et al., 2015; Zhang, 2015; Bian, 2016; Feng et al., 2016; Sheng and Wang, 2016; Wang et al., 2016; Wu et al., 2016; Zheng et al., 2016; Gao and Li, 2017; Li, 2017; Li et al., 2017; Ruan et al., 2017; Zeng W. Y. et al., 2017; Zeng Z. C. et al., 2017; Han et al., 2018; Liu et al., 2018; Luo, 2018; Shi et al., 2018) reported the therapeutic effects on BP as measured by SBP and DBP, including a total of 2,523 patients. We found that both SBP (*I^2^* = 95%, *P <*0.00001) and DBP (*I^2^* = 81%, *P <*0.00001) displayed great heterogeneity. We further conducted a subgroup analysis and selected a random-effects model to classify Western medicines for improving SBP into CCB (MD = −8.83, 95% CI [−11.22, −6.44], *P <* 0.00001), ACEI (MD = −6.45, 95% CI [−7.89, −5.01], *P <* 0.00001), ARB (MD = −12.01, 95% CI [−17.52, −6.51], *P <* 0.00001), and combined intervention (MD = −6.83, 95% CI [−11.96, −1.71], *P <*0.00001). Meanwhile, Western medicine for improving DBP was divided into CCB (MD = −1.07, 95% CI [−1.27, −0.86], P < 0.00001), ACEI (MD = −0.95, 95% CI −1.77 to −0.13, *P* = 0.02), ARB (MD = −12.01, 95% CI [−17.52, −6.51], *P <*0.00001), and combined intervention (MD = −6.83, 95% CI [−11.96, −1.71], *P <* 0.00001). Compared with Western medicine, combined application of CHM based on CCB, ACEI, ARB, and combined intervention could significantly reduce SBP and DBP (**Figures 4C, D**).

Sixteen trials reported (Xu et al., 2010; Shen et al., 2013; Zhu et al., 2013; Ou and Li, 2014; Lu et al., 2015; Bian, 2016; Sheng and Wang, 2016; Teng et al., 2016; Wang et al., 2016; Zheng et al., 2016; Gao and Li, 2017; Ruan et al., 2017; Liu et al., 2018; Luo, 2018; Sun, 2019) reported therapeutic effectiveness, including a total of 1,912 patients. The heterogeneity was low (*I^2^* = 0%, *P* = 0.89); thus, we selected a fixed-effect model to analyze the therapeutic effectiveness. The efficacy of CHM combined with Western medicine in treating hypertension was significantly higher than that of Western medicine alone. (RR = 1.21, 95% CI [1.16, 1.26], *P <* 0.00001; **Figure 4E**).

#### Vascular Endothelial Function

**Figure 5:**
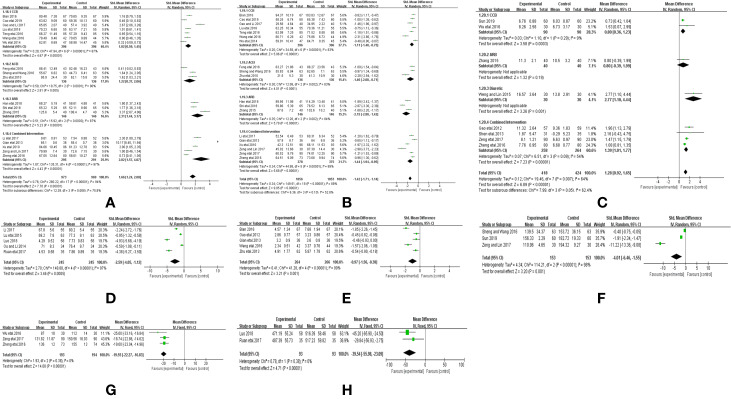
Forest plot of the comparison between CHM combined with conventional Western medicine and conventional Western medicine alone for **(A)** NO, **(B)** ET-1, **(C)** FMD, **(D)** VEGF, **(E)** hs-CRP, **(F)** Ang II, **(G)** vWF, and **(H)** TGFβ-1.

Eighteen trials reported the treatment effects on NO (Xu et al., 2010; Zhu et al., 2010; Qian et al., 2013; Wu et al., 2014; Zhang, 2015; Bian, 2016; Feng et al., 2016; Sheng and Wang, 2016; Teng et al., 2016; Wang et al., 2016; Zheng et al., 2016; Gao and Li, 2017; Li et al., 2017; Zeng Z. C. et al., 2017; Han et al., 2018; Liu et al., 2018; Shi et al., 2018; Cao et al., 2019), including a total of 1,942 patients. We found that NO had a large heterogeneity (*I^2^* = 94%, *P <* 0.00001). Then, we conducted a subgroup analysis and selected a random-effects model, and divided the Western medicine used to improve NO into CCB (SMD = 1.02, 95% CI [0.59, 1.45], *P <* 0.00001), ACEI (SMD = 1.22, 95% CI [0.37, 2.06], *P* = 0.005), ARB (SMD = 2.31, 95% CI [1.44, 3.17], *P <* 0.00001), and combined intervention (SMD =2.82, 95% CI [1.57, 4.07], *P <* 0.00001). Compared with Western medicine, combined application of CHM based on CCB, ACEI, ARB, and combined intervention could significantly improve the NO levels (**Figure 5A**).

Nineteen trials, including a total of 2,109 patients, reported the treatment effects on ET-1 (Xu et al., 2010; Zhu et al., 2010; Qian et al., 2013; Wu et al., 2014; Zhang, 2015; Bian, 2016; Feng et al., 2016; Sheng and Wang, 2016; Teng et al., 2016; Wang et al., 2016; Zheng et al., 2016; Gao and Li, 2017; Li et al., 2017; Zeng W. Y. et al., 2017; Zeng Z. C. et al., 2017; Han et al., 2018; Liu et al., 2018; Shi et al., 2018; Cao et al., 2019). ET-1 was associated with high heterogeneity (*I^2^* = 89%, *P <* 0.00001). We performed a subgroup analysis and selected random-effects model. The Western medicine used to improve ET-1 was divided into CCB (SMD = −1.11, 95% CI [−1.48, −0.75], *P <* 0.00001), ACEI (SMD = −1.40, 95% CI [−2.08, −0.71], *P <* 0.00001), ARB (SMD = −2.15, 95% CI [−2.88, −1.42], *P <* 0.00001), and combined intervention (SMD = −1.44, 95% CI [−1.94, −0.95], *P <* 0.00001). Compared with Western medicine, combined application of CHM based on CCB, ACEI, ARB, and combined intervention decreased ET-1 levels (**Figure 5B**).

Eight trials including a total of 842 patients reported treatment effects on FMD (Gao et al., 2012; Shen et al., 2013; Weng and Lin, 2015; Zhang, 2015; Wu et al., 2016; Zheng et al., 2016; Zeng W. Y. et al., 2017; Sun, 2019). FMD had a large heterogeneity (*I^2^* = 64%, *P* = 0.004); therefore, we performed a subgroup analysis and selected the random-effects model. The Western medicine used to improve FMD was divided into CCB (MD = 0.77, 95% CI [0.46, 1.07], *P <*0.00001), ARB (MD = 0.80, 95% CI [−0.39, 1.99], *P* = 0.19), diuretic (MD = 2.77, 95% CI [1.10, 4.44], *P* = 0.001), and combined intervention (MD =1.29, 95% CI [1.09, 1.49], *P <* 0.00001). Compared with Western medicine, combined application of CHM based on CCB, diuretic, and combined intervention could significantly improve FMD levels, but ARB combined with CHM showed no significant improvement (**Figure 5C**).

Five trials including a total of 490 patients reported treatment effects on VEGF (Ou and Li, 2014; Lu et al., 2015; Li, 2017; Ruan et al., 2017; Luo, 2018). As VEGF had a large heterogeneity (*I^2^* = 97%, *P <* 0.00001), we selected a random-effects model. As the number of trials was less than 10, subgroup analysis was not performed. Compared with Western medicine, CPAD could significantly reduce the VEGF levels (SMD = −2.59, 95% CI [−4.05, −1.12], *P* = 0.0005, **Figure 5D**).

Five trials including a total of 530 patients reported the treatment effects on hs-CRP (Gao et al., 2012; Qian et al., 2013; Zhu et al., 2013; Bian, 2016; Wang et al., 2016). As hs-CRP had a large heterogeneity (*I^2^* = 90%, *P <* 0.00001), we selected a random-effects model. As the number of trials was less than 10, subgroup analysis was not conducted. Compared with Western medicine alone, CPAD could greatly reduce the hs-CRP levels (SMD = −0.97, 95% CI [−1.56, −0.38], *P* = 0.001, **Figure 5E**).

Three trials including 306 patients reported treatment effects on Ang II (Sheng and Wang, 2016; Zeng Z. C. et al., 2017; Sun, 2019). Ang II was associated with high heterogeneity (*I^2^* = 98%, *P <* 0.00001). We selected random-effects model. As the number of trials was less than 10, subgroup analysis was not continued. Results revealed that compared with Western medicine, CPAD could reduce Ang II level (SMD = −4.01, 95% CI [−6.46, −1.55], *P* = 0.001, **Figure 5F**).

Three trials including a total of 387 patients reported the effects of treatment on vWF (Wu et al., 2016; Zheng et al., 2016; Zeng W.Y. et al., 2017). We found that the heterogeneity in vWF was low (*I^2^* = 0%, *P* = 0.38), and thus we selected a fixed-effect model. The results showed that compared with Western medicine alone, CPAD could significantly reduce the vWF levels (SMD = −19.55, 95% CI [−22.27, −16.83], *P <* 0.00001, **Figure 5G**).

Two trials including 186 patients reported the treatment effects on TGFβ-1 (Ruan et al., 2017; Luo, 2018). We found that its heterogeneity was low (*I^2^* = 0%, *P* = 0.38). We selected a fixed-effect model. The results showed that compared with the Western medicine alone, CPAD could significantly reduce the TGFβ-1 levels (SMD = −39.54, 95% CI [−55.98, −23.09], *P <* 0.00001, **Figure 5H**).

#### Adverse Event

Of all the included studies, four studies (Gao et al., 2012; Wu et al., 2014; Teng et al., 2016; Zheng et al., 2016) reported losses to follow, and nine (Wu et al., 2014; Ou and Li, 2014; Lu et al., 2015; Weng and Lin, 2015; Bian, 2016; Wu et al., 2016; Zeng Z. C. et al., 2017; Luo, 2018; Shi et al., 2018) studies reported adverse events. On initial recruitment, there were 3,265 patients whose blood pressures were measured. Altogether, 24 cases failed to follow-up and we finally collected 3,235 cases (99.09%) with complete data. Of nine studies reporting adverse events, five reported no adverse events during the study and three described the frequency of adverse effects in detail. The adverse reactions of the CPAD and control groups included edema of the lower extremity, flushing, and headache. All the reported adverse reactions were not aggravated and they all disappeared after symptomatic treatment.

### GRADE Evidence Profile

The GRADE evidence profile and summary of the findings are detailed in **Table 2**. Due to serious ROB in study methods, the heterogeneity and reporting bias, overall quality of evidence for 24 h-SBP, 24 h-DBP, SBP, DBP, NO, ET-1, and FMD were assessed as very low quality, low quality, indicating that these estimates were uncertain, and further studies are likely to influence our confidence when estimating CHM effects (**Table 2**).

**Table 2:** GRADEpro evidence grading.

Studies	Total (N)	Diagnosis standard	Intervention group			Control group			Treatment duration	Outcomes
			Sample size (M/F)	Age	Intervention	Sample size (M/F)	Age	Control		
Wu et al., 2014	137	Chinese guidelines published in 2005 and 2010 for the management of hypertension	47 (33/14)	47.58 ± 5.02	Bushen Qinggan decoction (*Gastrodia elata* Blume 30 g, *Uncaria rhynchophylla* (Miq.) Miq. 30 g, *Eucommia ulmoides* Oliv. 30 g, *Scutellaria baicalensis* Georgi 15 g, and bitter butyl tea 15 g) & Control	45 (29/16)	48.34 ± 4.25	amlodipine	8 weeks	24 h-SBP, 24 h-DBP, NO, ET-1
Qian et al., 2013	72	Chinese guidelines for the management of hypertention (2005)	36 (18/18)	66.0 ± 8.7	Jiangzhi Kangyanghua mixture (*Reynoutria multiflora* (Thunb.) Moldenke, *Crataegus pinnatifida* Bunge, *Forsythia suspensa* (Thunb.) Vahl, *Pueraria montana* var. *lobata* (Willd.) Maesen & S.M.Almeida ex Sanjappa & Predeep) & Control	36 (19/17)	65.8 ± 8.9	amlodipine + valsartan	8 weeks	SBP, DBP, NO, ET-1, hs-CRP
Gao et al., 2012	114	Chinese guidelines for the management of hypertention (2005)	57 (31/26)	68.42 ± 8.85	Yindanxinnaotong soft capsule [ginkgo leaves (0.5 g crude drug per capsule) (*Ginkgo biloba* L., Mant.); miltiorrhiza (0.5 g crude drug per capsule) (*Salvia miltiorrhiza* Bunge); herba erigeromtis (0.3 g crude drug per capsule) (*Erigeron breviscapus* (Vaniot) Hand.-Mazz.); gynostemma pentaphyllum (0.3 g crude drug per capsule (*Ginostemma pentaphyllum* (Thunb.) Makino); hawthorn (0.4 g crude drug per capsule)) *Crataegus pinnatifida* Bunge); allium sativum (0.4 g crude drug per capsule) (*Allium sativum* L.); panax notoginseng (0.2 g crude drug per capsule) (*Panax notoginseng* (Burkill) F.H.Chen); and borneol (0.01 g crude drug per capsule] & Control	59 (34/25)	67.69 ± 8.67	multiple antihypertensive drugs (CCB, ACEI/ARB, β-blocker, diuretic et al.)	6 months	SBP, DBP, FMD
Zhu et al., 2013	124	Chinese guidelines for the management of hypertention (2010)	62 (35 /27)	45.4 ± 6.75	Qingnao Jiangya tablets (*Scutellaria baicalensis* Georgi, *Prunella vulgaris* L., *Sophora japonica* L., Magnetitum, *Cyathula officinalis* K.C.Kuan, *Angelica sinensis* (Oliv.) Diels, *Rehmannia glutinosa* (Gaertn.) DC., *Salvia miltiorrhiza* Bge, *Whitmanian pigra* Whitman, *Gardenia jasminoides J.Ellis*, *Eucommia ulmoides* Oliv, *Senna obtusifolia* (L.) H.S.Irwin & Barneby, *Pheretima aspergillum* (E.Perrier), Hyriopsis cumingii (Lea)) & Control	62 (34/28)	44.9 ± 6.97	captopril	8 weeks	SBP, DBP, hs-CRP
Shen et al., 2013	66	internal medicine	31 (18/13)	60.13 ± 8.72	Tianma Gouteng granules (*Gastrodia elata* Blume *Uncaria rhynchophylla* (Miq.) Miq., *Concha haliotidis* 15 g, *Gardenia jasminoides* J.Ellis, *Eucommia ulmoides* Oliv. 10 g, *Scutellaria baicalensis* Georgi, *Cyathula officinalis* K.C.Kuan 20 g, *Leonurus cardiaca* L., *Taxillus chinensis* (DC.) Danser, *Reynoutria multiflora* (Thunb.) Moldenke, *Thespesia populnea* (L.) Sol. ex Corrêa) & Control	35 (22/13)	60.09 ± 12.13	multiple antihypertensive drugs (CCB, ACEI/ARB, β-blocker, diuretic et al.)	4 weeks	SBP, DBP, FMD
Ou and Li, 2014	72	Chinese guidelines for the management of hypertention (2006)	34 (17/17)	49.7 ± 8.9	Chinese Medicine of Resolving Phlegm and Dredging Collaterals (*Pinellia ternata* (Thunb.) Makino 15 g, *Citrus × aurantium* L. 15 g, *Thespesia populnea* (L.) Sol. ex Corrêa 10g, *Atractylodes macrocephala* Koidz. 10g, *Salvia miltiorrhiza* Bunge 10 g, *Leonurus cardiaca* L. 15 g, *Lycopus lucidus* var. *hirtus* (Regel) Makino & Nemoto 10 g, *Cyathula officinalis* K.C.Kuan 10 g, *Gastrodia elata* Blume 10 g, *Uncaria rhynchophylla* (Miq.) Miq.10 g, *Prunella vulgaris* L. 10 g, *Glycyrrhiza uralensis* Fisch. ex DC. 5 g) & Control	34 (18/16)	50.3 ± 9.0	multiple antihypertensive drugs (CCB, ACEI/ARB, β-blocker, diuretic et al.)	12 weeks	SBP, DBP, VEGF
Li et al., 2017	105	Chinese guidelines for the management of hypertention (2010)	53 (36 /19)	65.9 ± 5.3	Annao pill (*Bovis calculus artifactus*, *Sus scrofadomestica brisson*, *Cinnabari*s, *Borneolum syntheticum*, *Bubalus bubalis Linnaens*, *Pteria martensii* (Dunker), *Scutellaria baicalensis georgi*, *Coptis chinensis Franch*, *Gardenia jasminoides ellis*, *Realgar*, *Curcuma wenyujin* Y.H. Chen et C.Ling, *Gypsum fibrosum, haematitum*, *Hyriopsis cumingii* (Lea), *Mentha haplocalyx* Briq.) & Control	52 (31/21)	66.2 ± 5.2	multiple antihypertensive drugs (CCB, ACEI, aspirin, citicoline)	8 weeks	SBP, DBP, NO, ET-1
Liu et al., 2018	110	Chinese guidelines for the management of hypertention (2010) Chinese guidelines for the management of hypertention (2010)	55 (36 /19)	54.69 ± 5.81	Ziyin Huoxue decoction (*Cornus officinalis* Siebold & Zucc. 15 g, *Salvia miltiorrhiza* Bunge 15 g, *Angelica sinensis* (Oliv.) Diels 12 g, *Ligusticum striatum* DC. 12 g, *Chinemys reevesii (Gray)* 12 g, *Ligustrum lucidum* W.T.Aiton 12 g, *Anemarrhena asphodeloides* Bunge 12 g *Lycium barbarum* L. 12 g, *Rehmannia glutinosa* (Gaertn.) DC. 12 g (Praeparata), *Rehmannia glutinosa* (Gaertn.) DC. 10 g , *Paeonia lactiflora* Pall. 10 g, *Phellodendron chinense* C.K.Schneid 10 g.) & Control	55 (32/33)	53.21 ± 5.43	amlodipine besylate	8 weeks	SBP, DBP, NO, ET-1
Sheng and Wang, 2016	126	Chinese guidelines for the management of hypertention (2005)	63 (37/26)	69.78 ± 1.60	Bushen Huoxue decoction (*Rehmannia glutinosa* (Gaertn.) DC. 15 g, *Dioscorea japonica* Thunb. 10 g, *Cornus officinalis* Siebold & Zucc. 10 g, *Lycium barbarum* L. 10 g, *Eucommia ulmoides* Oliv. 10 g, *Achyranthes bidentata* Blume 10 g, *Angelica sinensis* (Oliv.) Diels 15 g, *Carthamus tinctorius* L. 10 g, *Conioselinum anthriscoides* ‘Chuanxiong' 10 g, *Ginkgo biloba* L. 10 g.) & Control	63 (35/28)	70.47 ± 1.58	multiple antihypertensive drugs (CCB, ACEI)	4 weeks	SBP, DBP, NO, ET-1, Ang II
Teng et al., 2016	170	Chinese guidelines for the management of hypertention (2010)	85 (50/35)	69.2 ± 5.9	Bushen Huoxue decoction (*Rehmannia glutinosa* (Gaertn.) DC. 30 g, *Lycium barbarum* L. 20 g, *Cornus officinalis* Siebold & Zucc. 10 g, *Alisma plantago-aquatica* subsp. *orientale* (Sam.) Sam. 10 g, *Thespesia populnea* (L.) Sol. ex Corrêa 10g, *Paeonia × suffruticosa* Andrews 10g, *Eucommia ulmoides* Oliv. 15 g, *Taxillus chinensis* (DC.) Danser 20g, *Apocynum venetum* L. 15 g, *Pinellia ternata* (Thunb.) Makino 10g*, Citrus × aurantium* L. 10 g, *Prunus davidiana* (Carrière) Franch. 10 g, *Carthamus tinctorius* L. 6 g, *Cyathula officinalis* K.C.Kuan 15 g, *Citrus × aurantium* L. 10 g, *Salvia miltiorrhiza* Bunge 20 g) & Control	85 (48/37)	69.8 ± 6.1	multiple antihypertensive drugs (CCB, ACEI)	12 weeks	24 h-SBP, 24 h-DBP, ET-1, NO
Gao and Li, 2017	80	Chinese guidelines for the management of hypertention (2010)	40 (19/21)	68.17 ± 3.28	Bushen Jieyu decoction (*Taxillus chinensis* (DC.) Danser 15 g, *Ligustrum lucidum* W.T.Aiton 15 g, *Bupleurum chinense* DC. 12 g, *Epimedium brevicornu* Maxim. 12 g, *Gastrodia elata* Blume 12 g, *Uncaria rhynchophylla* (Miq.) Miq. 15 g, *Angelica sinensis* (Oliv.) Diels 15 g, *Paeonia lactiflora* Pall. 12 g, *Thespesia populnea* (L.) Sol. ex Corrêa 15 g, *Atractylodes macrocephala* Koidz. 9 g, *Mentha canadensis* L. 6 g, *Glycyrrhiza uralensis* Fisch. ex DC. 6 g) & Control	40 (22/18)	68.52 ± 4.26	amlodipine besylate	8weeks	SBP, DBP, ET-1, NO
Wu et al., 2016	60	Chinese guidelines for the management of hypertention (2010)	30 (14/16)	68.16	Compound Qima capsule & Control	30 (15/15)	69.77	Nifedipine Controlled released Tablets	4 weeks	SDP, DBP, FMD, vWF
Li, 2017	110	Chinese guidelines for the management of hypertention	55 (30/25)	56.8 ± 8.9	Modified Wendan decoction (*Atractylodes lancea* (Thunb.) DC. 10 g, *Crataegus pinnatifida* Bunge 10 g, *Citrus × aurantium* L. 10 g, *Thespesia populnea* (L.) Sol. ex Corrêa 10 g, *Pinellia ternata* (Thunb.) Makino 10 g, *Atractylodes macrocephala* Koidz. 10 g, *Glycyrrhiza uralensis* Fisch. ex DC. 6 g, *Bambusa beecheyana* Munro 3 g) & Control	55 (28/27)	55.5 ± 8.5	amlodipine	12 weeks	SBP, DBP, VEGF
Lu et al., 2015	126	Chinese guidelines for the management of hypertention (2005)	63 (32/31)	59.73 ± 11.59	Modified Wendan decoction (*Atractylodes lancea* (Thunb.) DC. 10 g, *Crataegus pinnatifida* Bunge10 g, *Citrus × aurantium* L. 10 g, *Thespesia populnea* (L.) Sol. ex Corrêa 10 g, *Pinellia ternata* (Thunb.) Makino 10 g*, Citrus × aurantium* L. 10 g, *Atractylodes macrocephala* Koidz. 6 g, *Glycyrrhiza uralensis* Fisch. ex DC. 10 g, *Bambusa beecheyana* Munro 3 g) & Control	63 (33/30)	58.16 ± 10.97	AmlodiieMaleateTalet	12 weeks	SBP, DBP, VEGF
Wang et al., 2016	86	Chinese guidelines for the management of hypertention	42 (27/15)	54.19 ± 12.48	Pinggan Qianyang decoction (*Sigesbeckia glabrescens* (Makino) Makino 15 g, *Prunella vulgaris* L. 15 g, *Concha haliotidis* 15 g, *Styphnolobium japonicum* (L.) Schott 15 g, *Gastrodia elata* Blume 15g, *Scutellaria baicalensis* Georgi 15 g, *Salvia miltiorrhiza* Bunge 15 g, *Cyathula officinalis* K.C.Kuan 10 g, *Taxillus chinensis* (DC.) Danser 10 g, *Plantago asiatica* L. 15 g) & Control	44 (26/18)	53.48 ± 12.37	amlodipine besylate	6 months	SBP, DBP, hs-CRP, NO, ET-1
Feng et al., 2016	86	WHO/ISH Guidelines for the Treatment of Hypertension (1999)	43 (30/13)	62.75 ± 1.42	Qiwei Tiaoya granules (*Gastrodia elata* Blume 1.5 g, *Uncaria rhynchophylla* (Miq.) Miq. 3 g, *Concha haliotidis* 3.6 g, *Eucommia ulmoides* Oliv. 2.4 g, *Arctium lappa* L. 1.8 g, *Testudinis carapax et plastrum* 3 g, *Carapax trionycis* 3 g), Zhibai Dihuang Pills & Control	43 (30/13)	61.37 ± 3.84	Perindopril Tablets	8 weeks	SBP, DBP, ET-1, NO
Cao et al., 2019	120	Chinese guidelines for the management of hypertention (2010)	60 (33/27)	58.68 ± 9.37	Jianpi Tongluo decoction (*Astragalus mongholicus* Bunge 15 g, *Thespesia populnea* (L.) Sol. ex Corrêa 15 g, *Pinellia ternata* (Thunb.) Makino 10 g, *Pheretima aspergillum*(E.Perrier) 12 g, *Salvia miltiorrhiza* Bunge 12 g, *Carthamus tinctorius* L. 10 g, *Conioselinum anthriscoides* ‘Chuanxiong' 10 g) & Control	60 (31/29)	57.48 ± 8.58	amlodipine besylate	4 weeks	24 h-SBP, 24 h-DBP, ET-1, NO
Zeng W. Y. et al., 2017	180	Chinese guidelines for the management of hypertention (2010)	90 (48/42)	56.17 ± 6.73	Quyu Huoxue decoction (*Uncaria rhynchophylla* (Miq.) Miq. 30 g, *Citrus × aurantium* L. 15 g, *Thespesia populnea* (L.) Sol. ex Corrêa 15 g, *Salvia miltiorrhiza* Bunge 15 g, *Paeonia lactiflora* Pall. 15 g, *Gastrodia elata* Blume 15 g, *Alisma plantago-aquatica* subsp. *orientale* (Sam.) Sam. 15 g, *Leonurus cardiaca* L. 15 g, *Pinellia ternata* (Thunb.) Makino 10 g) & Control	90 (51/39)	56.33 ± 6.80	multiple antihypertensive drugs (CCB, ARB, diuretic)	3 months	SBP, DBP, FMD, ET-1, vWF
Zheng et al., 2016	160	Chinese guidelines for the management of hypertention (2010)	80 (45/35)	68.1 ± 7.9	Songling Xuemaikang capsule (*Pinus pinea* L., *Pueraria lobate* (Willd).Ohwi, *Pteria martensii* (Dunker)), Qiju Dihuang Pill (*Rehmannia glutinosa* (Gaertn.) DC., Cornus officinalis Sieb.et Zucc., *Dioscotea opposita* Thunb. Paeonia suffruticosa Andr. Poria cocos (Schw.) Wolf, Alisma orientale (Sam.) Juzep., Anemarrhena asphodeloides Bge., Phellodendron chinense Schneid.) & Control	80 (47/33)	67.5 ± 7.2	antihypertensive drugs (CCB, ARB, diuretic)	12 weeks	SBP, DBP, FMD, NO, ET-1, vWF,
Han et al., 2018	82	Chinese guidelines for the management of hypertention (2010)	41 (23/18)	69.8 ± 3.15	Danshen Dripping pills (*Salvia miltiorrhiza* Bge., *Panax notoginseng* (Burk.) F.H.Chen,, *Borneolum syntheticum*) & Control	41 (25/16)	69.51 ± 3.14	Telmisartan	12 weeks	SBP, DBP, NO, ET-1
Zeng Z. C. et al., 2017	60	Chinese expert consensus on the diagnosis and treatment of hypertension in the elderly	30 (17/13)	49.80 ± 6.45	Tianma Gouteng decoction (*Gastrodia elata* Blume 20 g, *Uncaria rhynchophylla* (Miq.) Miq. 10 g, *Concha haliotidis* 15 g, *Gardenia jasminoides J.Ellis* 10 g, *Eucommia ulmoides Oliv*. 10 g, *Scutellaria baicalensis Georgi* 10 g, *Cyathula officinalis K.C.Kuan* 20 g, *Leonurus cardiaca* L. 10 g, *Taxillus chinensis* (DC.) Danser 15 g, *Reynoutria multiflora* (Thunb.) Moldenke 10 g, *Thespesia populnea* (L.) Sol. ex Corrêa 15 g) & Control	30 (9/21)	71.97 ± 7.82	multiple antihypertensive drugs (CCB, ARB)	8 weeks	SBP, DBP, Ang II, NO, ET-1
Sun, 2019	120	Chinese guidelines for the management of hypertention (2010)	60 (33/27)	58.17 ± 9.25	Tianma Gouteng decoction (*Gastrodia elata* Blume 10 g, *Uncaria rhynchophylla* (Miq.) Miq. 15 g, *Concha haliotidis* 15 g, *Gardenia jasminoides* J.Ellis 30 g, *Eucommia ulmoides* Oliv. 10 g, *Scutellaria baicalensis* Georgi 15 g, *Cyathula officinalis* K.C.Kuan 15 g, *Leonurus cardiaca* L. 15 g, *Taxillus chinensis* (DC.) Danser 15 g, *Reynoutria multiflora* (Thunb.) Moldenke 30 g, *Thespesia populnea* (L.) Sol. ex Corrêa 15 g)	60 (37/23)	58.95 ± 8.43	felodipine	3 months	24 h-SBP, 24 h-DBP
Weng and Lin, 2015	60	Chinese guidelines for the management of hypertention (2010)	30 (17/13)	49.80 ± 6.45	Tianma Gouteng decoction (*Gastrodia elata* Blume 9 g, *Uncaria rhynchophylla* (Miq.) Miq. 9 g, *Concha haliotidis* 9 g, *Gardenia jasminoides* J.Ellis 18 g, *Eucommia ulmoides* Oliv. 9 g, *Scutellaria baicalensis* Georgi 9 g, *Cyathula officinalis* K.C.Kuan 9 g, *Leonurus cardiaca* L. 12 g, *Taxillus chinensis* (DC.) Danser, *Reynoutria multiflora* (Thunb.) Moldenke 9 g, *Thespesia populnea* (L.) Sol. ex Corrêa 9 g) & Control	30 (15/15)	52.30 ± 5.37	hydrochlorothiazide	12 weeks	24 h-SBP, 24 h-DBP, FMD
Bian, 2016	134	Chinese guidelines for the management of hypertention (2010)	67 (43/24)	56.6 ± 8.3	Tianma Gouteng decoction (*Gastrodia elata* Blume 20 g, *Uncaria rhynchophylla* (Miq.) Miq. 15 g, *Concha haliotidis* 15 g, *Gardenia jasminoides* J.Ellis 15 g, *Eucommia ulmoides* Oliv. 15 g, *Scutellaria baicalensis* Georgi 15 g, *Cyathula officinalis* K.C.Kuan 15 g, *Leonurus cardiaca* L. 15 g, *Taxillus chinensis* (DC.) Danser 15 g, *Reynoutria multiflora* (Thunb.) Moldenke 10 g, *Thespesia populnea* (L.) Sol. ex Corrêa 10 g) & Control	67 (43/24)	58.3 ± 8.7	levamlodipine besylate	8 weeks	SBP, DBP, hs-CRP, NO, ET-1
Xu et al., 2010	189	WHO/ISH Guidelines for the Treatment of Hypertension (1999)	96 (52/44)	53.0 ± 8.9	Tianma Huangqin pills (*Gastrodia elata* Blume, *Scutellaria baicalensis* Georgi) & Control	93 (40/53)	51.2 ±7.8	multiple antihypertensive drugs (CCB, ACEI/ARB, β-blocker, diuretic et al.)	6 weeks	SBP, DBP, 24 h-SBP, 24 h-DBP, ET, NO
Shi et al., 2018	130	Chinese expert consensus on the diagnosis and treatment of hypertension in the elderly	65 (34/31)	72.8 ± 4.3	Tianzhi decoction (*Gastrodia elata* Blume 10 g, *Uncaria rhynchophylla* (Miq.) Miq. 30 g, *Whitmania pigra Whitman* 6 g, *Pueraria montana* var. *lobata* (Willd.) Maesen & S.M.Almeida ex Sanjappa & Predeep 30 g, *Senna obtusifolia* (L.) H.S.Irwin & Barneby 20 g, *Achyranthes bidentata* Blume 20 g, *Prunella vulgaris* L. 10 g, *Paeonia lactiflora* Pall. 15 g, *Angelica sinensis* (Oliv.) Diels 10 g, *Chrysanthemum × morifolium* (Ramat.) Hemsl. 10 g) & Control	65 (36/29)	71.6 ± 3.7	irbesartan	4 weeks	SBP, DBP, ET, NO
Ruan et al., 2017	70	Chinese guidelines for the management of hypertention (2010)	35 (20/15)	42.10 ± 4.98	Tongmai Huazhuo decoction (*Carthamus tinctorius* L. 20 g, *Pinellia ternata* (Thunb.) Makino 15 g, *Salvia miltiorrhiza* Bunge 30 g, *Thespesia populnea* (L.) Sol. ex Corrêa 30 g, *Typha orientalis* C.Presl 15 g, *Crataegus pinnatifida* Bunge 25 g, *Raphanus raphanistrum* subsp. *sativus* (L.) Domin 20 g) & Control	35 (18/17)	40.98±5.00	amlodipine besylate	8 weeks	SBP, DBP, TGFβ-1, VEGF
Zhang, 2015	80	Chinese guidelines for the management of hypertention (2005)	40 (23/17)	51.6 ± 7.2	Xuefu Zhuyu decoction (*Prunus davidiana* (Carrière) Franch. 12 g, *Carthamus tinctorius* L. 10 g, *Angelica sinensis* (Oliv.) Diels 12 g, *Rehmannia glutinosa* (Gaertn.) DC. 10 g, *Conioselinum anthriscoides* ‘Chuanxiong' 5 g, *Paeonia lactiflora* Pall. 10 g, *Achyranthes bidentata* Blume 10 g, *Platycodon grandiflorus (*Jacq.) A.DC. 5 g, *Bupleurum chinense* DC. 3 g, *Citrus × aurantium* L. 5 g, *Glycyrrhiza uralensis* Fisch. ex DC. 3 g) & Control	40 (25/15)	52.3 ± 8.4	candesartan cilexeti	8 weeks	SBP, DSP, NO, ET -1, FMD
Luo, 2018	116	Chinese guidelines for the management of hypertention (2010)	58 (33/25)	69.1 ± 8.4	Yiqi Huoxue Tongluo decoction (*Astragalus mongholicus* Bunge 50 g, *Pheretima aspergillum*(E.Perrier) 15 g, *Salvia miltiorrhiza* Bunge 15 g, *Cyathula officinalis* K.C.Kuan 15 g, *Crataegus pinnatifida* Bunge 15 g, *Angelica sinensis* (Oliv.) Diels 10 g, *Paeonia lactiflora* Pall. 10 g, *Prunus davidiana* (Carrière) Franch. 6 g, *Carthamus tinctorius* L. 6 g, *Cinnamomum cassia* (L.) J.Presl 6 g*, Glycyrrhiza uralensis* Fisch. ex DC. 6 g) & Control	58 (35/23)	68.8±8.5	multiple antihypertensive drugs (CCB, ARB, diuretic)	4 weeks	SBP, DSP, TGFβ-1, VEGF
Zhu et al., 2010	90	Chinese guidelines for the management of hypertention (2005)	30 (19/11)	51.32 ± 5.73	Yishen Pinggan decoction (*Eucommia ulmoides*Oliv., *Taxillus chinensis* (DC.) Danser, *ramulus Uncaria rhynchophylla (*Miq.) Miq., *Apocynum venetum* L., *Pueraria montana* var. *lobata* (Willd.) Maesen & S.M.Almeida ex Sanjappa & Predeep lobatae, *Cyathula officinalis* K.C.Kuan) & Control	30 (21/9)	53.21 ± 5.43	benazepril	3 months	24 h-SBP, 24 h-DBP, ET, NO

### Publication Bias

**Figure 6:**
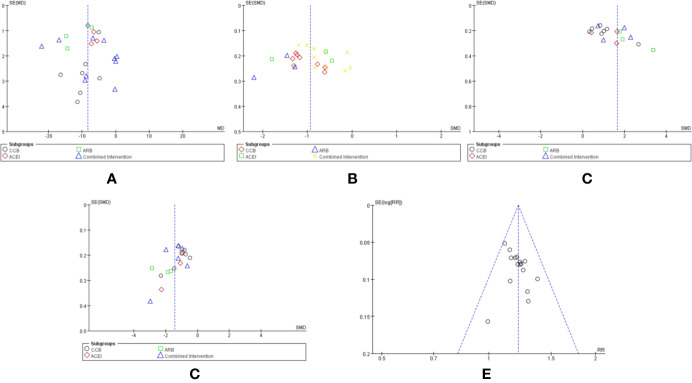
Funnel plot of the comparison between CHM combined with conventional Western medicine and conventional Western medicine alone for SBP **(A)**, DBP **(B)**, NO **(B)**, ET-1 **(D)**, and therapeutic effects **(E)**.

Funnel plot analysis for the outcomes of SBP (A), DBP (B), NO (C), ET-1 (D), and therapeutic effects (E) was performed to explore the publication bias. The funnel plot was asymmetric, suggesting a mild publication bias in this systematic review (**Figure 6**).

## Discussion

TCM herbal formulas have always been recommended as complementary and alternative treatments for hypertension in China and other countries (Xiong et al., 2018). With the concerns of long-term medication and adverse reactions of antihypertensive drugs, some mild to moderate hypertensive patients who are not willing to take antihypertensive drugs would prefer CHM either used alone or in combination with antihypertensive drugs. In their opinion, TCM was efficacious in improving symptoms, reducing fluctuations in BP, improving vascular endothelial function associated with hypertension, and reducing the amount of conventional Western medicine despite its bitter and slightly sweet taste (Wu et al., 2014). In addition, as CHM has been used for thousands of years, it seems to be relatively safe (Xiong et al., 2015). However, whether TCM is beneficial for hypertension on vascular endothelial function is not well recognized until now. To our knowledge, this is the first systematic review and meta-analysis of the published RCTs to sum up the effects of TCM for hypertension treatment on vascular endothelial function and provide the latest level of evidence for patients, policymakers, and clinicians.

### Summary of Main Results

As compared with the conventional Western medicines, the results of this meta-analysis showed marked improvements in BP and vascular endothelial function for hypertensive patients treated with CPAD, although there was some heterogeneity among these studies. NO and ET are essential substances synthesized and secreted mainly by endothelial cells for dilatation and vascular contraction, respectively, and their levels in blood were used to evaluate vascular endothelial injury (Zhong et al., 2011). FMD is a conventional assessment of conduit artery function with great cardiovascular insight (Broxterman et al., 2019). Meta-analysis in this study proves that CPAD could obviously protect vascular endothelial function in hypertensive patients by increasing the serum NO levels, reducing that of ET, and improving FMD. Hypertension is also related to endothelial dysfunction. Hypoxia induced by vascular endothelial injury is one of the most important factors in inducing VEGF expression. VEGF is an important mediator of the Ang II-induced inflammatory reaction of vasculature. It releases VEGF, attracts circulating neutrophils and monocytes, and increases the production of inflammatory mediators, leading to hypertension (Zhao et al., 2004). VEGF and other inflammatory markers are greatly elevated in hypertensive patients, especially in patients with uncontrolled BP, and VEGF levels are directly related to the levels of SBP and CRP (Marek-Trzonkowska et al., 2015). VWF and TGFβ-1 are also vital indicators for the evaluation of vascular endothelial injury (Ryan et al., 2003; Kido et al., 2008). This meta-analysis showed that compared with conventional Western medicines, remarkable improvements were displayed in VEGF, hs-CRP, vWF, and TGFβ-1 for hypertensive patients treated with CPAD. We conducted a subgroup analysis of the above results with more than 10 studies. The antihypertensive drugs were divided into CCB, ACEI, ARB, diuretics, and combination drugs. The results showed that CHM combined with CCB and combined intervention could significantly improve 24 h-SBP, 24 h-DBP, SBP, DBP, NO, ET-1, and FMD. CHM combined with ACEI could remarkably improve SBP, DBP, NO, ET-1, but failed to reduce 24 h-SBP and 24 h-DBP. CHM combined with ARB could greatly improve SBP, DBP, NO, and ET-1, but failed to increase FMD. CHM combined with diuretics could obviously increase FMD but failed to reduce 24 h-SBP and 24 h-DBP. Moreover, the completion rate of all studies was more than 99% without severe adverse events, indicating that CHM might be an effective and safe choice for hypertension by alleviating symptoms and improving the well-being of hypertensive patients.

### Strengths and Limitations

In clinical practice, antihypertensive Western medicines have a clear curative effect, but all of them have certain side effects. They cannot be completely eliminated from clinical use, and it is impossible to avoid situations where some patients may not be willing to take Western drugs and may not fully meet the needs of clinical treatment for hypertension. TCM classic herbal formulas with fixed herbs, definite curative effects, and fewer adverse effects for certain diseases have been practiced since ancient times (Xiong et al., 2017). Meanwhile, TCM classic herbal formulas have been recommended as complementary and alternative treatments in China and other countries. Thus, in Asian countries, some hypertensive patients have turned to TCM treatment with fewer adverse effects (Xiong et al., 2013). A previous meta-analysis demonstrated that a TCM adjuvant to antihypertensive drugs might be beneficial for hypertensive patients for lowering BP, improving depression, regulating blood lipids, and inhibiting inflammatory responses (Xiong et al., 2015; Chen et al., 2018; Xiong et al., 2018; Huang et al., 2019; Xiong et al., 2019). However, these studies do not focus on vascular endothelial function injury, which is one of the most common reactions of hypertension and plays a vital role in the occurrence and development of hypertension. The combination of CHM and Western medicine for essential hypertension treatment has become a trend in East Asia. At present, there is no definite index for evaluating the function of the vascular endothelium. To comprehensively evaluate the function of the vascular endothelium, we included many blood indicators to ensure that the results are comprehensive. The evaluation of the vascular endothelial function has not been included as one of the important factors affecting the cardiovascular prognosis of patients in the latest hypertension treatment guidelines. Moreover, there are no drugs specifically for vascular endothelial injury. The analysis in this study may provide some evidence for further improvement of the treatment guidelines.

After quantitative synthesis, our review is the first to demonstrate that CPAD can lower BP and improve vascular endothelial function in patients with hypertension, suggesting that TCM as an adjuvant therapy could be used for hypertension treatment by alleviating symptoms and improving the well-being.

However, there are some limitations to our review. We only conducted a search based on Chinese and English studies, and it is possible that articles related to CPAD for hypertension may have been published in other languages. Moreover, in this systematic review, we did not consider differences in the composition and dosage of CPAD and the course of medication, which may have some influence on treatment efficacy. The methodological quality of the included trials was quite low, except that CPAD was difficult to blind. The included studies had other flaws, including poor randomization and allocation concealment. According to the GRADE system, the evidence of CPAD for hypertension treatment was assessed to be of very low, low, or moderate quality. Therefore, evidence supporting CPAD in the treatment of patients with hypertension was inconclusive.

### Implications for Research

In this review, CPAD may be effective and safe for the treatment of hypertension, but the methodological quality of the included studies was poor, evidence for efficacy and safety was insufficient, and clinical heterogeneity was large; therefore, great attention should be paid when interpreting current evidence and potential findings. Further research is needed to evaluate the efficacy and safety of CPAD for treating hypertension. Rigorous RCTs with large sample sizes and high-quality methodologies are required to explore the efficacy of CPAD in clinic and provide evidence-based data for promoting the use of CPAD.

## Conclusion

To summarize, our meta-analysis suggests that compared with conventional Western medicine alone, CPAD might be effective in reducing BP levels and improving vascular endothelial function in patients with hypertension. As there are some methodological limitations to the studies included, these findings are required to be interpreted carefully. To further strengthen this evidence, new, well-designed studies with large sample sizes, strict randomization, and detailed descriptions about the detection and reporting processes are warranted.

## Data Availability Statement

All datasets generated for this study are included in the article/supplementary material.

## Author Contributions

LH, MW, and WR: Conceived and designed the experiments. WR, JL, LL, DY, and RY: Performed the experiments. RQ, LL, and JL: Searched the literature. WR, LH, and RY: Data extraction and risk of bias assessments. WR, MW, and LL: Analyzed the data. WR: Wrote the paper. WR, MW, JL, LL, DY, RY, and LH: Read and approved the manuscript.

## Funding

This work was supported by the National Natural Science Foundation of China (no. 81573777) and the Beijing Municipal Natural Science Foundation (no. 7162172). The funders had no role in the study design, data collection and analysis, decision to publish, and preparation of the manuscript.

## Conflict of Interest

The authors declare that the research was conducted in the absence of any commercial or financial relationships that could be construed as potential conflict of interest.

The reviewer YS declared a shared affiliation, with no collaboration, with several of the authors, WR, DY, MW, RY, and LL, to the handling editor at the time of review.

## Abbreviations

CHM, Chinese herbal medicine; ROB, Risk of bias; CVD, Cardiovascular disease; DALYs, Death- and disability-adjusted life-years; BP, Blood pressure; b-blocker, Beta-adrenergic blocking agents; ACEI, Angiotensin-converting enzyme inhibitors; ARB, Angiotensin II receptor blockers; CCB, Calcium channel blockers; TCM, Traditional Chinese Medicine; CAM, Complementary and alternative medicine; CHM, Chinese herbal medicine; CPAD, CHM plus antihypertensive drugs; PRISMA, Preferred Reporting Items for Systematic Reviews and Meta-Analyses; SBP, Systolic blood pressure; DBP, Diastolic blood pressure; NO, Nitric oxide; FMD, Flow mediated dilation; VEGF, Vascular endothelial growth factor; hs-CRP, Highsensitivity C-reactive protein; Ang II, Angiotensin II; vWF, von Willebrand Factor; TGFb-1, Transforming growth factor b-1; RCTs, Randomized controlled trials; RR, Relative risk; CI, Confidence interval; MD, Mean difference; SMD, Standard mean difference.
